# Sharpening Working Memory With Real-Time Electrophysiological Brain Signals: Which Neurofeedback Paradigms Work?

**DOI:** 10.3389/fnagi.2022.780817

**Published:** 2022-03-28

**Authors:** Yang Jiang, William Jessee, Stevie Hoyng, Soheil Borhani, Ziming Liu, Xiaopeng Zhao, Lacey K. Price, Walter High, Jeremiah Suhl, Sylvia Cerel-Suhl

**Affiliations:** ^1^Lexington Veteran Affairs Medical Center, Lexington, KY, United States; ^2^College of Medicine, University of Kentucky, Lexington, KY, United States; ^3^Department of Mechanical, Aerospace, and Biomedical Engineering, University of Tennessee, Knoxville, Knoxville, TN, United States; ^4^New Mexico Veteran Affairs Medical Center, Albuquerque, NM, United States

**Keywords:** closed-loop feedback, brain computer interface (BCI), working memory, EEG-ERPs, BCI illiteracy, biofeedback

## Abstract

Growing evidence supports the idea that the ultimate biofeedback is to reward sensory pleasure (e.g., enhanced visual clarity) in real-time to neural circuits that are associated with a desired performance, such as excellent memory retrieval. Neurofeedback is biofeedback that uses real-time sensory reward to brain activity associated with a certain performance (e.g., accurate and fast recall). Working memory is a key component of human intelligence. The challenges are in our current limited understanding of neurocognitive dysfunctions as well as in technical difficulties for closed-loop feedback in true real-time. Here we review recent advancements of real time neurofeedback to improve memory training in healthy young and older adults. With new advancements in neuromarkers of specific neurophysiological functions, neurofeedback training should be better targeted beyond a single frequency approach to include frequency interactions and event-related potentials. Our review confirms the positive trend that neurofeedback training mostly works to improve memory and cognition to some extent in most studies. Yet, the training typically takes multiple weeks with 2–3 sessions per week. We review various neurofeedback reward strategies and outcome measures. A well-known issue in such training is that some people simply do not respond to neurofeedback. Thus, we also review the literature of individual differences in psychological factors e.g., placebo effects and so-called “BCI illiteracy” (Brain Computer Interface illiteracy). We recommend the use of *Neural modulation sensitivity* or BCI insensitivity in the neurofeedback literature. Future directions include much needed research in mild cognitive impairment, in non-Alzheimer’s dementia populations, and neurofeedback using EEG features during resting and sleep for memory enhancement and as sensitive outcome measures.

## Significance of Working Memory and Improvement

Most of us have experienced the “tip of the tongue” feeling of not quite recalling a name, a face, or items from memory. As part of normal brain and cognitive aging, such “senior moments” increase, especially under stress. The lure of brain training is the possibility to directly enhance neural activities associated with good memory and therefore reduce the moments of memory lapses. A central component of cognitive ability is working memory, i.e., the capacity to hold active information in one’s memory for immediate manipulation. By definition, working memory shares a large amount with other core cognitive functions (e.g., cognitive control and attention), which are strongly associated with performance in intelligence tests (Chen et al., 2019). Measurements of neural activity have become strong predictors of mild cognitive impairments in persons with various kinds of cognitive deficits. Electrophysiological changes during working memory are some of the earliest signals in preclinical risk of mild cognitive impairment (Jiang et al., 2021).

Neurofeedback (NF) is biofeedback that uses real-time sensory reward for brain activity associated with certain performance (e.g., accurate and fast recall). In contrast, traditional cognitive rehabilitation typically applies offline behavioral reward (e.g., money). Growing evidence supports the idea that the ultimate biofeedback is to reward with sensory pleasure (e.g., enhanced visual clarity) in real-time to neural circuits that are associated with a desired performance, such as excellent memory (YuLeung To et al., 2016) and attention (deBettencourt et al., 2015, 2019). Using functional neuroimaging, brain regions that have been consistently implicated in neurofeedback or real-time self-modulation include the anterior insula (cognitive control, self-awareness), and the basal ganglia (sensory motor integration, implicit learning), independent of the targeted region-of-interest (Emmert et al., 2016). There are different networks involved in neurofeedback in cognitive and affective functions (Trambaiolli et al., 2021); this is seen particularly in fMRI-based studies that allow better spatial definition of the structures involved during cognition and affective processes (Paret et al., 2019; Skottnik et al., 2019; Direito et al., 2021).

## Why Focus on Real-Time Electroencephalography-Based Neurofeedback

Since fMRI-based (blood-and-oxygen-level dependent) signals are hundreds of times slower than electrophysiological signals, there is a strong case to be made for the advantages of using electrophysiological signals (Ros et al., 2013, 2014) measured by methods such as electroencephalography (EEG) or magnetoencephalography (MEG). In addition to high temporal resolution, human scalp EEG is non-invasive, affordable, with no movement restriction, and is more suitable to large-scale applications. EEG is a tool that measures the summations of neural postsynaptic potentials at the scalp. EEG-based NF has been successful in enhancing attention in older adults (Jiang et al., 2017). Using a double-blind controlled design in the older brains, seminal work by Angelakis et al. (2007) applied EEG NF in an older population and showed improved processing speed and executive functions. Additional success has been reported using EEG-based NF training in older dementia patients (Surmeli et al., 2016).

Despite exciting progress using NF in brain training to improve memory in young and older adults, most of the NF training studies thus far have been limited to traditional frequencies. NF training using event-related potentials (ERPs), i.e., averaged EEG signals associated with cognitive events, is currently lacking in the literature.

So, once the field gains new knowledge of neuromarkers of specific neurophysiological functions, then neurofeedback training should be better targeted beyond the single frequency approach. The new methods should include frequency interactions and event-related potentials. The challenges include both neurological understanding as well as technical difficulties for real-time closed-loop feedback (Sitaram et al., 2017).

Very few studies have applied memory-related potentials directly in the context of preclinical and clinical Alzheimer’s pathology. ERPs are a well-studied approach for indexing brain responses associated with memory and cognition. These electrophysiological outcome measures can predict individual risk of mild cognitive impairment 5 years before diagnosis (Jiang et al., 2021).

Neurofeedback has been used to improve cognitive and physical performance of humans (Daly and Wolpaw, 2008; Pfurtscheller et al., 2008; Machado et al., 2013; Broccard et al., 2014; Chaudhary et al., 2016). While the efficacy of these methods differs, all have been reported to enhance performance by training attention (another core function of cognition) and working memory tasks. However, evidence for improvement in everyday life utilizing cognition is limited, which provides the impetus for developing better and time-efficient methods to directly train neural processes underlying attention. A closed-loop BCI system can be designed to directly control external devices. Also, it can be utilized as a neurofeedback platform in neurorehabilitation programs to improve and enhance the cognitive abilities (Chaudhary et al., 2016).

While there is fundamental disparity between neurofeedback and BCI (Abiri et al., 2019, 2020), combining both approaches can potentially help to expand achievements of each individual approach.

For instance, the goal in motor-imagery BCIs is to control an external object by inducing and modulating the brainwaves of interest during the training session so that the BCI system can determine the user’s intention in real-time in testing sessions. Users must learn how to regulate their brainwaves which then makes the BCI analogous to neurofeedback. In both, users must learn to modulate their brain activity to acquire a certain feedback, specific output, or greater reward. The similarity may result in triggering similar cognitive and neural processes. Thus, the training process of BCI may expand our knowledge of neurofeedback.

## In Search of Better Rewards in Neurofeedback Systems

Most of the neurofeedback studies have utilized a few channels of EEG signals or a fixed and narrow range of frequencies (Gruzelier, 2014a,b) while most of the BCI systems, such as motor-imagery BCIs have incorporated multiple EEG channels and individualized and adaptable ranges of frequencies determined by signal processing, machine learning, or deep learning approaches. Embracing those approaches in neurofeedback regiments may help to distinguish and extract individualized features from EEG signals with which we may obtain higher efficacy compared to having a fixed channels and fixed frequency approach (Ang et al., 2012).

There is also research about task-unspecific internal and external confounding variables characterized and studied in the BCI literature. For instance, the attentional state of the user has been quantified with the level of mu and gamma bands in different brain areas. The measure has helped to enumerate the level of attention and motivation in a motor-imagery BCI task (Grosse-Wentrup et al., 2011). Having those measures alongside the neurofeedback therapy can be beneficial to assess how likely a user is motivated to participate in neurofeedback therapy and how one neurofeedback regiment may be adapted and accepted by the users over another one.

The type and form of feedback signal is another instrumental element in neurofeedback. We have five main senses: visual, auditory, somatosensory, olfactory, and gustatory. Although vision is the most prominent sensory feedback in humans compared to other primates, other sensory feedbacks may play a significant role in learning and modulating our brainwaves in response to different phenomena.

There are yet more challenges to applying neurofeedback. One challenge is how to define the reinforcement signals. It might be easier and ethically more appropriate to define the reinforcement signal in non-human experiments. For example, withholding food from a rat and providing it later as a reward when the animal successfully modulates a neurophysiological signal may not be an ethical concern (at least now), but it would be more challenging and clearly unethical to deprive human subjects of food. Another challenge is that there is no guarantee that a human subject would interpret the feedback as a reward. So, the motivational state of the subject results in different interpretation of the feedback signal.

Clinical neurofeedback training has been shown to be an effective treatment for people with a wide range of deficits, including epilepsy (Sterman and Egner, 2006; Strehl et al., 2014), attention deficit hyperactivity disorder (ADHD) (Arns et al., 2009), stroke (Rayegani et al., 2014), autistic spectrum disorder (ASD) (Kouijzer et al., 2009), emotional disorders (Reiter et al., 2016), and tinnitus (Hartmann et al., 2014). Furthermore, neurofeedback has been evaluated as a means to enhance cognitive control in healthy people (Zoefel et al., 2011; Wang and Hsieh, 2013; Gruzelier, 2014a,b). The quality and quantity of changes (effect size) due to the neurofeedback regimen can be enumerated with behavioral responses, measured by cognitive scoring questionnaires, or demonstrated by neural measures of cognition.

## Electroencephalography, Event-Related Potential Neurofeedback in Improving Cognitive Complaints and Impairment

The basic EEG components in human adults are the delta, alpha, theta, beta, and gamma frequency bands. Alpha frequency during eyes-closed resting EEG has emerged as an important biomarker in mild cognitive impairment and dementia (Babiloni et al., 2021a; Cecchetti et al., 2021; (Stoiljkovic et al., 2021). Alpha oscillations (8–12 Hz), sourced in frontal sites including the anterior cingulate cortex, are related to working memory and related performance in humans. This EEG wave is the most common wave seen during awake states (Adrian and Matthews, 1934). Global alpha power is more abnormal in younger MCI and AD patients (Babiloni et al., 2021b). Posterior alpha sources are more abnormal in male MCI/AD patients (Babiloni et al., 2021c).

The EEG theta rhythm (4–8 Hz) is also related to memory performance. Theta rhythms have been shown to play a role in encoding episodic memories and are also correlated with behavioral performance (Hasselmo and Stern, 2014). EEG delta rhythms (0.5–3.5 Hz) are somewhat different than the other three as an increase in delta band activity leads to inhibition (Harmony, 2013). This inhibition is useful however, as it blocks interference from other centers in the brain, allowing one to concentrate on the mental task at hand. Delta waves are prominent in deep stages of sleep. Finally, beta rhythms (13–35 Hz) are related to brain consciousness and motor functions. Both alpha and beta waves are common during awake states, but beta waves are also present in states of drowsiness (Nayak and Anilkumar, 2020). Finally, gamma rhythms (30–80 Hz) are higher frequency EEG rhythms that have demonstrated a role in visual and sensory processing. Due to the low amplitude of the gamma rhythms, these EEG frequencies have been historically difficult to measure but have recently been linked to other rhythms and functions. However, rapidly growing new literature on gamma rhythms and its underlying biological basis is fascinating (e.g., Milekovic et al., 2019). NF using high frequency components are a new frontier needing research.

Increasing evidence suggests the coupling of two different rhythms is also used to perform specific functions. Coupling of the brain’s theta-gamma rhythms (θ–γ) has demonstrated a link between the cortical regions (prefrontal areas and the cingulate cortex) which plays a major role in working memory (Schack and Klimesch, 2002). Delta-theta coupling has been shown to be important in decision making and cognitive processing (Adams et al., 2019). Interestingly, changes in theta-gamma coupling have been shown to be earlier neuromarkers than traditional AD biomarkers. AD and MCI patients demonstrate the lowest level of θ–γ coupling in a verbal working memory task in comparison with healthy participants (Goodman et al., 2018). An impairment of θ–γ coupling that increases in parallel to the progression of the MCI has been recently reported in human patients (Musaeus et al., 2020). These new findings suggest that coupling of brain oscillations is critical for proper cognitive functioning and will likely be another neuromarker for applying NF treatment.

## What Neurofeedback Paradigms of Working Memory Worked and Did Not Work?

To determine the most successful way of performing a NF study, samples of previous studies of successful and unsuccessful studies were analyzed. Twelve studies involving older adults (> 60 years old) and eight involving younger adults (< 35 years old) were selected for using several different parameters with varied success reported in each study. Investigating the differences seen between different age groups, however, provided important insight into the quality of studies performed in the past. As much of human cognitive development occurs before the age of twelve, there is a rational fear that once a body reaches a certain stage, mental growth and training is impossible. Although there is increasing evidence that the brain remains plastic past its “peak years,” the physiological degeneration due to brain aging is an obstacle to training older adults with NF (Mattson and Arumugam, 2018).

After a review of successful past studies, it was demonstrated that the aging brain can in fact be trained and improved, despite the anatomic and pathological limitations faced. There were seven studies involving younger individuals investigated, five of which were successful in using NF to train the brain. Of the eleven studies involving patients over sixty, nine were successful in training the brain with NF, two of which involved patients with MCI. This demonstrates that not only can the healthy aging brain be trained successfully with NF, but a degenerating aging brain can be trained as well. Although there are many different factors that potentially change the results in these studies, it is promising to see evidence that the aging brain can still be changed and improved upon, as this provides an avenue into treating more severe diseases such as Alzheimer’s disease. The factors that potentially confound these studies are compared below.

Each study used a slightly different method in controlling for their BCI group, so it was important to investigate the different controls used (Nicolas-Alonso and Gomez-Gil, 2012; Sorger et al., 2019). Between the studies that used older and younger populations, there were three common types of control groups used: a “waitlist” control, a sham NF control, and a group that received cognitive training in place of the NF (some used a combination of these or no control at all, but these were the most common occurring groups). The waitlist control groups received no training between the first and last testing sessions. The sham NF groups received the NF training sessions but did not receive the NF protocol to change any specific EEG band. The cognitive training groups used other cognitive training methods such as brain teasers and memory games in place of the NF. Control groups will be discussed in much more detail later in this review.

Two primary modalities used in the NF studies were investigated: visual and auditory. A majority of the studies utilized a visual NF protocol. In the projects that used visual feedback (Krause et al., 2017), a visual reward such as a motivating progress bar or pleasing image was given upon activating the specific EEG band. The auditory feedback was similar except it used a pleasing tone for achieving the goal range and an unpleasant tone for missing the EEG power range. The combination study was unsuccessful at achieving its goal. The fifteen visual modality studies were successful where one out of the two auditory modality studies were successful. More research needs to be performed to determine whether auditory only NF protocols are useful in training the brain, as two studies are not a large enough sample size to draw a conclusion.

Follow up time was the major limitation found throughout each study included in this review. Only one study reported longitudinal findings (Marlats et al., 2020). This group had participants return and re-test after 1 month. They demonstrated that scores for several different memory tasks remained elevated from preliminary testing after receiving NF training. Follow up is vital to the integrity of any study like this as it demonstrates the clinical power EEG NF training can have on those with declining cognitive function. Having at least a follow up after 1 month should be an integral portion of any NF study moving forward; continuing follow ups through the year will increase the power of the studies by showing prolonged cognitive improvements.

Another component to the follow up that requires consideration is whether there is a need for re-training. Spaced repetition is a useful technique for memorizing large amounts of information and could also be a useful tool when considering the long-lasting effects of EEG based NF. Doing a certain number of sessions throughout a year could prove to have even more benefits than performing 10–20 sessions within the span of 1 month. This, as well as the vitality of the training during follow up sessions, will need to be investigated in the future.

## Individual Differences in Neurofeedback

Neurofeedback did not work in some studies listed in Tables 1, 2. We investigated individual differences in brain signals for answers. Strong individual differences, including but not limited to genetic and environmental differences, have been reported in working memory and performance (Parasuraman and Jiang, 2012), and how cognitive training effects are preserved and learning is transferred (Greenwood and Parasuraman, 2015). Learning transfer means in cognitive training in one domain (working memory) can transfer in similar tasks (short-transfer) and untrained tasks (long-transfer). However, there are strong individual differences in applying learning transfer to important daily tasks (Sinotte and Coelho, 2007; Westerberg et al., 2007; Cicerone et al., 2011; Kuo et al., 2014). Attention, mood, and motivation are important factors (Kadosh and Staunton, 2019).

**TABLE 1:** Sample NF training in older adults with or without success.

References	Experiment design	EEG band*	Modality/location	Goals	Main findings	Did it work? (Y/N)
Lavy et al., 2021	NF training/sham control (12 sessions)	Upper alpha	Visual/Pz	Treat patients with MCI by using NF to increase the upper alpha frequency at the central parietal electrode.	Significant improvement in cognitive ability was demonstrated following NF training. This improvement was sustained over the following 30 days.	Y
Reis et al., 2016	NF with cognitive training/Cognitive training only/NF sham (8 sessions, 30 min)	Alpha + /Theta +	Visual/FL (avg. frontal left), FR, PL, PR	Preservation of cognitive function in healthy aging patients	NF with cognitive training showed more improvements than the cognitive training only group while also increasing alpha and theta bands. NF only group showed similar improvements.	Y
Jirayucharoensak et al., 2019	NF group (32 aMCI/26 healthy)/Game group (aMCI/17 healthy)/Care as usual group (14 aMCI/11 healthy) – 20 sessions	Alpha/Beta	Visual/Global	Enhance cognitive performance in patients with MCI *via* a game-based NF system	NF improves sustained attention and spatial working memory, but had no effect on pattern recognition memory and short term visual memory which are hallmarks of MCI.	Y
Bielas and Michalczyk, 2021	NF training/No NF control (20 sessions)	Beta +	Visual/Cz	Test the behavioral effects of beta NF training in attentional control in the elderly	Significant improvement was seen in the NF group on the Simon and Stroop tests where the control group did not see significant results.	Y
Gomez-Pilar et al., 2016	NF training/control (5 sessions)	Beta +	Visual/C3, Cz, C4	Test motor imagery based BCI program to enhance cognitive function related to old age.	Significant cognitive improvements were seen in visuospatial, oral language, memory, and intellect after 5 NF training sessions.	Y
Wang and Hsieh, 2013	Theta NF training (old and young)/NF sham control (12 sessions)	“Frontal midline Theta activity uptraining”	Visual/30 different electrodes	Investigate theta uptraining protocol on attention and WM of both young and older patients	Both young and old training groups had increases in attention when compared to the control and the older group showed increased memory.	Y
Becerra et al., 2011	NF to increase memory/Sham NF (30 sessions, 30 min each)	Theta-	Auditory/F4, C3, P4, F7, T5	Reduced Theta	Experimental group showed greater improvement in EEG and behavioral measures, but control group also showed small improvements in memory.	Y
Marlats et al., 2020	No control listed (20 sessions, twice per week for 10 weeks, 45 min)	SMR (sensori motor rhythm)/Theta	Visual/Auditory/Cz	Improve cognitive decline in elderly patients with MCI	Theta and alpha power during eyes closed resting state showed significant improvement after 1 month follow up and scores improved for different memory tasks (MoCa, RAVLT, WAIS, Forward digit span)	Y
Campos da Paz et al., 2018	NF training/Sham NF/No NF control (10 sessions, 30 min)	SMR +	Visual/Cz	Test if SMR protocol can improve WM performance in aging population	SMR NF improved visual working memory performance after the training for training group only. Alpha and beta frequency bands were increased at frontal and temporal regions.	Y
Wang and Hsieh, 2013	Theta NF training (old and young)/NF sham control (12 sessions)	“Frontal midline Theta activity uptraining”	Visual/30 different electrodes	Investigate theta uptraining protocol on attention and WM of both young and older patients	Both young and old training groups had increases in attention when compared to the control and the older group showed increased memory.	Y
Lee et al., 2015	Training group/Waitlist control (24 session, 30 min)	All ranges	Not NF	Increase cognitive ability in the elderly with a BCI training method	Training group scored higher on post-test than waitlist one (not great study though)	Y
Staufenbiel et al., 2013	Gamma + = Higher cognitive score Beta + = Higher familiarity scores (8 sessions, 30 min)	Beta + /Gamma +	Auditory, Fz used for NF	Increase overall cognition of the elderly through beta/gamma NF	Gamma and Beta frequencies were increased, but no cognitive performance changes were observed	N
Lecomte and Juhel, 2011	NF training/non-NF relaxion/waiting list control (4 sessions, 1 h)	Alpha/Theta ratio +	Auditory (eyes closed) and Visual (eyes open)/C3, Cz, C4	Improve short term memory performance	NF increased alpha/theta frequency ratio but showed no improvement of memory performance.	N

*An increase or decrease in EEG band power is indicated with “+” or “–” respectively.

**TABLE 2:** Sample NF training in young adults with or without success.

References	Experiment/control	EEG Band*	Modality/Location	Goal	Main finding	Did it work? (Y/N)
Wei et al., 2017	Alpha NF group/control (12 sessions, 5 blocks of 5 min per session)	Alpha +	Visual (on smartphone app)/	Working memory and episodic memory was tested with an at home EEG NF device that increases alpha rhythm.	A portable NF system was able to successfully train a significant increase in alpha power as well as significantly enhance accuracy of both working and episodic memory tasks.	Y
Bagherzadeh et al., 2019	Right neurofeedback training group > trained to increase alpha power in right vs. Left neurofeedback training group > trained to increase alpha power in left	Alpha +	Visual (screen)	Test for a casual role of alpha synchrony in attention	There is an “association between alpha symmetry and covert spatial attention in that covertly attending to one hemifield led to increase in alpha in the ipsilateral hemisphere and decreases in alpha in the contralateral hemispheres” Also, higher alpha power in the left compared with right parietal cortex in the LNT group had increased “visually evoked responses and attentional bias toward the stimuli in ipsilateral visual field”—opposite was true for RNT group	Y
Hsueh et al., 2016	NF with alpha/NF with random frequency (12 sessions, ∼45 min)	Alpha +	Visual/C3, Cz, C4	Improvement in all memory tasks through alpha NF training	Working memory and episodic memories showed significant increases alongside alpha power increases in the NF training group but not in the control.	Y
Escolano et al., 2011	NF training/control (5 sessions, 30 min each)	Upper alpha +	Visual/F3, Fz, F4, C2, Cz, C4, P3, Pz, P4, O1, Oz, O2	Evaluate reliability of upper alpha NF training effects and to enhance working memory performance. Also to do passive open eyes resting state.	UA frequency band was increased during active tasks independent of other frequency bands while significantly improving working memory when compared to control group.	Y
Zoefel et al., 2011	NF training/control (5 sessions, 30 min each)	Upper alpha +	Visual/P3, Pz, P4, O1, O2	Improved cognitive performance with increased upper alpha frequency	Upper alpha frequency increases and improved cognitive performances were seen only in the NF training group and not in the control.	Y
Xiong et al., 2014	NF training/Behavioral training/sham NF/no training control (12 sessions)	Theta/alpha ratio +	Visual/Whole brain	Upregulating the theta/alpha power ratio to increase working memory in healthy young adults	Normal young adults succeeded in improving their WM performance with EEG NF and was significantly greater than the control groups.	Y
Escolano et al., 2011	NF training/control (5 sessions, 30 min each)	Upper alpha +	Visual/F3, Fz, F4, C2, Cz, C4, P3, Pz, P4, O1, Oz, O2	Evaluate reliability of upper alpha NF training effects and to enhance working memory performance. Also to do passive open eyes resting state.	UA frequency band was increased during active tasks independent of other frequency bands while significantly improving working memory when compared to control group.	Y
Gordon et al., 2019	NF + WMT/NF + ACT/NF/WMT/ACT/control (6 groups) – 10 sessions	Upper alpha +	Visual/Pz	EEG NF improves inhibition and working memory in healthy young adults	WMT and NF + WMT groups showed improvements in both upper alpha band frequency and cognitive performance but did not have significant improvements of scores compared to the silent control group.	N
Jurewicz et al., 2017	NF to increase beta/NF to decrease beta (16 sessions)	Beta (±)	Visual/P3, P4, F3, F4	EEG NF manipulation of beta bands in healthy young adults to improve attention	Although attention was not shown to increase in relation to changing beta band, there were unintentional alterations of the alpha band implicating that alpha is more prone to manipulation through EEG-NF	N

*An increase or decrease in EEG band power is indicated with “+” or “–” respectively.

### Placebo Effects

A placebo is a treatment with no positive or negative side effects. The placebo effect is a phenomenon where a patient’s condition improves even though they were given a treatment that has no mechanism to alleviate their symptoms. Finniss et al. (2010) provide psychological explanations for the placebo effect. First, the patient expecting their symptoms to abate can be enough to lead to the placebo effect. One example of the placebo effect is an experimenter telling the patient the medication will cause a reduction in headaches leads to an actual reduction in the patient’s headaches because they expect the medication to work. Another is classical conditioning, where if a patient’s headache gets relieved by the medication the first-time they take it, they will expect that to happen every time. The combination of expectancy and classical conditioning can cause a significant placebo effect, creating a challenge for scientists to conclude if the medication was truly helpful or if it was the placebo effect (Finniss et al., 2010). One neurofeedback study showed participants with a positive expectancy of the treatment had a decrease in symptoms while participants with a negative expectancy of the treatment had an increase in their symptoms (Lee and Suhr, 2020).

For researchers to elucidate if their treatment is helpful or if the participant is experiencing placebo effect results, they often do double blind studies. Double blind studies are structured so neither the participant nor the experimenters know who receives the real treatment and who receives the placebo. Designing an experiment this way allows adequate comparison to help draw a conclusion regarding true efficacy or a placebo effect. Neurofeedback characteristics cause difficulties in designing an effective double-blind experiment because the automatic rewards a participant receives during a double-blind study elicit a different response than the rewards manually programmed into a single-blind neurofeedback study (Lansbergen et al., 2010). The single-blind experiment leads to an increased possibility for experimenter bias. Confounding variables like a participant getting coaching or getting rewarded for focusing further complicates the experiment’s design (Eugene Arnold et al., 2020).

### Bioethical Debates in Brain Training

The NF and double-blind studies have shown effectiveness in a variety of treatments e.g., psychiatric disorders (Mehler et al., 2018; Dudek and Dodell-Feder, 2021), children’s ADHD (Steiner et al., 2014; Riesco-Matías et al., 2021), chronic pain (Patel et al., 2020). They have also dealt with diseases that ranged from Parkinson’s disease to cognitive decline in stroke victims. Is it ethical to withhold treatment that could vastly improve a participant’s quality of life? Sandler and Bodfish (2008) told children diagnosed with ADHD and their parents that they were going to be given a placebo. The children’s symptoms remained constant when given the placebo and a lower dose ADHD medication but worsened when they only received the lower dose ADHD medication. This finding may mean that researchers can run more ethical placebo neurofeedback studies by making sure the participants are fully aware and agree to the possibility of receiving a placebo.

A deficiency in the studies in Tables 1, 2 was a lack of placebo and/or double-blind methodology. Table 3 consists of studies that did contain a placebo and/or double-blind methodology. Although Table 3 depicts potential benefits for older adults, only two experiments in the table used a double-blind study, meaning that it is possible there were some expectancy results in the experiments (Mottaz et al., 2018; Nicholson et al., 2020). There is hope for older adults suffering from mild cognitive impairments, strokes, and Parkinson’s disease that neurofeedback may be a safe and effective treatment for them. Further studies need to be done, particularly double-blind studies, to prove the positive outcomes of neurofeedback treatment and eliminate confounding variables. Many of the placebo tests did not have a follow up, so it is impossible to tell if the positive outcomes of the neurofeedback will be retained long term. The same limitation was found in Tables 1, 2. Mottaz et al. (2018) did have a follow up and saw an improvement in their participants’ functional connectivity (FC) over the controls’. The improvement did not last during a follow-up investigation.

**TABLE 3:** NF with control in working memory and cognitive motor.

References	Participant age	Experiment/control	EEG band*	Modality	Goal	Did it work?	Length of Trials/Follow up	Main Findings
Campos da Paz et al., 2018	Average was 69.05 years old	NF training vs. sham NF training vs. no NF training	SMR (sensorimotor rhythm) +	Visual–19 Channels	To test if SMR Protocol can help improve working memory performance in older adults	Yes—to an extent	10 training sessions, twice per week, for 5 weeks	There was a significant improvement in the NF group and the no NF group. However, the sham group also improved, possibly showing that the act of training alone helped improve working memory.
Wang and Hsieh, 2013	Mean age for older NF group—65 years; mean age for younger adult is 21 years,	NF training in younger and older adults vs. placebo NF in younger and older adults	Frontal Midline Theta + , rEEG	Visual—Whole Brain (32 sites)	To test if uptraining theta activity could help improve attention and working memory	Yes—to an extent	3 times per week for 4 weeks	Both NF groups improved over their respective sham groups. Working memory was significantly improved in the older adult NF group. Therefore, using NF to upregulate frontal midline theta, may help with cognitive aging.
Mottaz et al., 2018	Mean age—57.1 years old	Double blind study, cortex FC training vs. control region FC training	Alpha, rEEG FC	Visual—Whole Brain	To see if functional connectivity (FC) had an effect on behavioral motor performance in stroke patients	Yes—to an extent	Two sessions per week over the course of a month for a total of eight sessions, for both the control and NF group	The cortex FC training did elicit improvements in motor function over the FC control, however, there was not long-term retention
Nicholson et al., 2020	Age range—21–59 years	Double blind—experimental group vs. sham control group	Alpha -, rEEG FC	Visual—Pz, fMRI	To see if downregulating alpha waves can help reduce PTSD symptoms and to further investigate the default mode network (DMN) involvement in PTSD.	Yes	Weekly sessions for a 20 week periods with a 3 month clinical follow up, sham control did not receive NF sessions	The PTSD severity scores were lower in the experimental group vs. the sham control. The experimental group also showed a normalization of DMN and SN connectivity.
Subramanian et al., 2011	Age Range- 39–75 years old	Experimental group vs. control group	Supplementary motor area (SMA) +	Motor Imagery—fMRI, whole brain	To see if SMA + NF can improve motor function in patient with Parkinson’s Disease	Yes	2 fMRI scan session with 2 runs of NF 2–6 months apart, with a behavioral follow up two weeks after the 2nd scan session	The experimental group increased their SMA and had an improvement in motor symptoms. The control group did not experience these effects.

*An increase or decrease in EEG band power is indicated with “+” or “-” respectively.

*SMR, sensorimotor rhythms; FC, functional connectivity.*

### Brain Computer Interface Insensitivity

Brain-Computer Interface (BCI) illiteracy describes participants in neurofeedback studies who do not adequately achieve performance goals. Due to negative connotations surrounding “illiteracy,” “BCI insensitivity” is proposed instead to describe the phenomenon of non-respondence (Sannelli et al., 2019). “BCI insensitivity” describes participants unable to train successfully as well as participants incapable of performing neurofeedback with the desired accuracy. It is estimated that BCI inefficiency can be found in 15–30% of neurofeedback participants (Blankertz et al., 2010). Researchers can divide participants into three groups—participants who can be successfully trained with appropriate accuracy, participants who can be successfully trained but did not reach the required accuracy during neurofeedback, and participants who could neither be successfully trained nor achieve the required accuracy during neurofeedback (Vidaurre and Blankertz, 2010).

Neurofeedback can be a costly and time-consuming treatment. Because up to 30% of participants may not receive the desired effects from neurofeedback, researchers look for potential predictors in participants that may have BCI inefficiency. Hammer et al. (2012), found participants who had higher fine motor skills and a better ability to self-regulate focus performed better during neurofeedback training. Lower peaks of sensory motor rhythm (SMR) may be indicative of less likelihood of a participant achieving successful neurofeedback (Sannelli et al., 2019). Participants’ initial BCI performance may also predict their overall outcome or how much training a participant will need to be successful (Neumann and Birbaumer, 2003; Kübler et al., 2004). To help save time and money in studies, Blankertz et al. (2010) designed a program to predict a participant’s performance based on a 2-min resting EEG with eyes opened.

As more predictors of BCI insensitivity are published, the question then becomes, is it ever possible for participants to achieve neurofeedback success with BCI insensitivity? The answer is yes. Vidaurre and Blankertz (2010) developed a coadaptation calibration program that allowed users to increase their SMR peaks, increasing BCI performance. Sannelli et al. (2016) developed an adaptive learning model using common spatial patterns and sensory motor rhythms that helped participants who failed to achieve performance goals in prior neurofeedback studies. Methods involved visual evoked potentials and tactile sensation have also been explored in BCI (Yao et al., 2018; Volosyak et al., 2020). Going forward, researchers may want to use a combination of the predictive techniques along with techniques for improving BCI efficiency to help participants achieve the best results. Furthermore, more BCI studies involving older adults are needed for researchers to make adequate conclusions for BCI training in different age groups.

The studies in Table 3 were chosen based on several different criteria. Only studies that had a control/placebo group were included. Experiments that primarily used children and young adults were excluded as the focus of this paper is on older adults. Experiments in Table 4 were chosen based on the criteria that the researchers were specifically looking at BCI with the use of neurofeedback. Unfortunately, due to the limited number of studies using controls, this table could not be limited to only experiments with control groups. The same is true for only using experiments that were mainly comprised of older adults.

**TABLE 4:** Brain-computer Interface (BCI) training in healthy adults and patients.

References	Participant age	Experiment/control	EEG band	Modality	Goal	Did it work?	Length of trials/follow up	Main findings
Vidaurre and Blankertz, 2010	N/A	Three groups: One group was successful in NF and Training; One NF did not work; One group where neither training nor NF helped	SMR	Visual, Motor- C3, Cz, C4	To successfully train those with BCI	yes	8 feedback runs with 100 trials over the course of 1 day	It was possible for participants to gain BCI control with this technique that couldn’t gain control before
Sannelli et al., 2016	N/A	No control, mainly used previous participants who did not have success with neurofeedback. They had to train through three adaptive levels	Common Spatial Pattern, SMR, rEEG	Visual, Motor- C3, Cz, C4, CFC4, PCP2, CP3, central areas and mastoid references	To successfully trained those with BCI	yes	Five runs of NF, no follow up	The CSPP (common spatial pattern patches) method allowed users who had previously not had success with neurofeedback to train faster with better neurofeedback success, potentially reducing BCI inefficiency.
Sannelli et al., 2019	Mean age 29.9 years, with a range of 17–65 years	Three groups were tested—A group where training and NF were successful, A group where training was successful, but NF did not help, and finally a group where neither training nor NF helped	SMR, CSP, LAP, Beta, rEEG	Visual, Motor: C3, C4	Gain a better understanding of SMR BCI	Some possible predictors of BCI inefficiency were found	2 sessions, psychological test on day 1, BCI training with 10 EEG recordings and a short psychological exam on day 2	The height or absence of an SMR peak can help predict BCI performance
Neumann and Birbaumer, 2003	Age range 31–66 years	No control, Five paralyzed patients with ALS	Slow Cortical Potentials	Visual—Fz, Cz, and Pz	To study S effect on BCI performance	To an extent	Different amount of training depending on performance (8–32 training days)	The participant’s initial performance could help predict their future SCP performance
Kübler et al., 2004	Mean age of healthy patients was 26, mean age of ALS patients was 50.6	Healthy patients vs. patients with ALS	SCP	Visual—Cz	To try and find a predictor of BCI success in ALS patients	Yes	2–12 daily sessions	The initial phase performance can predict the amount of training a participant will need to reach satisfactory performance
Yao et al., 2018	Average was 22 years old with a range of 19–42	Tactile Sensation vs. Motor Imagery	Upper and Lower Alpha, Beta	Virbotactile Stimulation, motor imagery—Whole Brain	To find a potential method for decreasing BCI in NF experiments	Yes	Tactile sensation group had 80 sessions, while the motor imagery experiment group had 63 sessions	The tactile sensation group performed better than the motor imagery group. The tactile sensation method could be used in the future to decrease BCI.
Volosyak et al., 2020	“Typical ages of University Students”	Compare BCI performance by using steady-state visual evoked potentials (SSVEPs), Steady-state motion visual evoked potentials (SSmVEP) and code-modulated Visual Evoked Potentials (cVEPS)	Visually evoked potentials (VEPs)	Audio, visual—Pz, P3, P4, P5, P6, PO3, PO4, PO7, PO8, POO1, POO2, O1, O2, O9, O10, Cz, and AFz	To find a method for decreasing BCI in NF experiments and look for personal preferences and demographic factors in BCI performance	yes	Each participant completed three sessions, one session for each paradigm being tested.	The VEPs examined in this study did not illicit BCI illiteracy. There was not a performance difference in males vs. females.
Blankertz et al., 2010	29.9 years old	No control	SMR, CSP, rEEG (w/eyes open)	Motor Imagery- C3, C4	To propose a program that could predict BCI performance	Yes	225 motor imagery trials	The researchers were able to develop a program that used a 2 min open eyed rEEG to predict BCI performance.
Hammer et al., 2012	Average age—29.5 years old, age range was 17–65 years	No control, participants completed multiple psychological tests as well as one session of neurofeedback	SMR, rEEG	C3, C4	To find psychological predictors of BCI performance	Yes	three runs of 100 trials	Participants with better fine motor skills had fewer mistakes. Participants that had higher concentrate abilities are performed better.

## The Theoretical Basis of Brain Training of Working Memory With Electroencephalography Neurofeedback Treatment

Cognitive aging is caused by synaptic, metabolic, and structural changes during brain aging which slowly lead to loss of full cognitive function. EEG measures synchronized synaptic functions and network at the scalp. Recent work has identified neurosynaptic changes as one of the earliest biomarkers of preclinical AD, appearing before onset of tau-mediated neuronal injury or brain structure changes (Jack et al., 2011; Sperling et al., 2011). Among the most common early symptoms of dementia are deficits in working memory. The exact neural mechanisms subserving working memory are under debate (Mi et al., 2017). There are three types of hypotheses: First, the *Frequency model* suggested that WM involves periodic reactivation of memory representations at each gamma cycle within gamma-theta nested oscillations in the hippocampus, mediated by slow after depolarizations with a time constant that should be matched to the theta period. Assuming each memory is activated exactly once during a theta burst cycle, the same memories are repeatedly reactivated over subsequent theta cycles (Lisman and Idiart, 1995; Siegel et al., 2009). Deficits in higher frequency such as gamma rhythms are associated with cognitive deficits. Therefore, brain stimulation studies (Chan et al., 2021; Mimenza-Alvarado et al., 2021), applying gamma frequency treatment in mild cognitive impairment and mild Alzheimer’s disease, set foundations for future neurofeedback applying gamma rhythm.

The question remains why manipulating lower frequencies such as delta and alpha improves working memory performance. New evidence suggests that glial astrocyte reactivity and Ca + channel related slow waves altered in the brains of persons with dementia might be the cause of the low frequency alterations (Sardinha et al., 2017). Astrocytic signaling supports hippocampal-prefrontal theta synchronization and cognitive function (Lenk et al., 2020).

Second, the persistent firing model hypothesizes that working memory is supported by persistent activity of certain neurons active in the prefrontal cortex, hippocampus, parietal, and other cortical and subcortical networks, in the absence of direct perceptual stimulations. For instance, enhanced EEG activity during memory retrieval is observed in frontal sites, which are different from visually evoked potentials during perception. The attractiveness of this model is that it explains the memory network supporting working memory functions *via* synchronized oscillations at the same frequency in different brain regions. Recent evidence in animal models and with neuroimaging also points to brain connectivity networks as novel neuro-markers for indexing early deficits in AD risk. Aß peptides disrupt neural activity at the synaptic level and induce aberrant activity patterns in neural network circuits within and between brain regions in animal models (Palop and Mucke, 2010). Patterns of functional brain connectivity in humans are highly predictive of cognitive performance (Hachinski et al., 2006; Finn et al., 2015). The blood-oxygen-dependent level brain signals measured by functional MRI show that brain connectivity (particularly bilateral parietal and frontal-temporal) correlates with CSF AD biomarkers (Aß and tau), particularly during working memory tasks (Jiang et al., 2016). Overall synaptic synchronization during an intrinsic spontaneous resting state measured by EEG output has also been linked to neuroinflammation (astrocytes reactivity and calcium signaling; Wang et al., 2021), which provides a potential treatment direction.

Third, *Synaptic theory of working memory* is a synaptic-based theory for short-term information storage in neural circuits (Mongillo et al., 2008; Lundqvist et al., 2011). In this model, memory is retained by an item-specific pattern of synaptic facilitation. This mechanism does not require neurons to fire with elevated rates for the whole duration of the memory task, resulting in a robust and metabolically more efficient scheme (Mi et al., 2017). Resting EEG and network oscillations are correlated to working memory performance (Borhani et al., 2019) and cognitive impairment (McBride et al., 2013, 2014, 2015).

Finally, the Neurofeedback to frontal and pre-frontal cortices enhance goal-directed behaviors and executive functions. The goal-directed behaviors are strongly associated with the brain activity in the prefrontal cortex (Miller and Cohen, 2001). Executive functions encompass the mental processes that allow individuals to take control over automatic responses of the brain to produce goal-oriented behaviors (Garon et al., 2008). The prefrontal cortex functions comprise planning, goal setting, decision making, voluntary attention, task switching, set shifting, behavioral and perceptual inhibitions, voluntary regulation, and error correction. In therapeutical neurofeedback, especially the ones that target cognitive control, some of the functions seem to be fundamental to set up intrinsic reward, to integrate feedback information, and to self-regulate behavior. Most of those functions interact with attention, a broad concept that can be defined as the set of processes dealing with the allocation of working memory to the different neural representations available in the brain (Knudsen, 2007). Attention plays a critical role in allocating the brain resources to working memory while high-level cognition relies on working memory to learn tasks (Cowan et al., 2005). There are studies indicating a shared neural mechanism that supports both attention and working memory (Ikkai and Curtis, 2011; Gazzaley and Nobre, 2012). The shared neural mechanisms support the theory that there is also a mutual connection between the two brain functions. Thus, event-related (memory and/or attention) brainwave patterns should be targets of NF training.

## Neurofeedback Training Beyond Electroencephalography Frequency Bands: Event-Related Potential-Based Neurofeedback

Electroencephalography recordings directly measure post-synaptic potentials. Recording the averaged EEG signals, i.e., event-related potentials (ERP), during cognitive events known as cognitive ERP is a promising but less often investigated approach for indexing brain mechanisms underlying cognition and memory (Olichney et al., 2008, 2011, 2013; Li et al., 2017; Jiang et al., 2021). Memory-related neuromarkers are sensitive to general cognitive decline before conventional biomarkers of AD can be detected by CSF/PET methods and behavioral performance changes.

Using longitudinal follow-up of healthy older adults over 10 years, Jiang et al. (2021) revealed that the different brainwaves between working memory responses to Targets and Non-targets in three left frontal electrodes (F7, F5, or F3) or averaged from three left frontal sites are the best predictor for diagnosis of cognitive impairment 5 years before diagnosis. Intriguingly, memory non-targets or distractors did not predict well.

**FIGURE 1:**
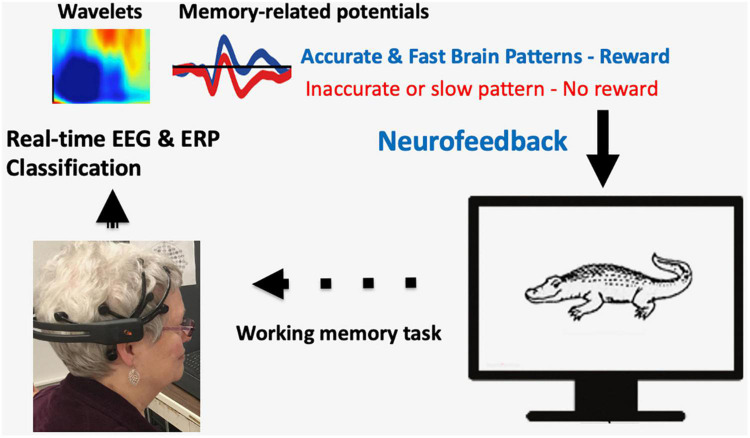
A sample illustration of closed-loop Neurofeedback training for memory improvement. A participant wearing a wireless EEG headset performs a visual memory task. Recorded EEG signals are analyzed in real-time, e.g., EEG wavelet analysis (frequency, power, and time) or ERP memory-related potentials. Neurofeedback is given by rewarding brain patterns that are associated with accurate and fast memory retrieval.

A participant wearing a wireless EEG headset performs a visual memory task (Figure 1). Recorded EEG signals are analyzed in real-time, e.g., EEG wavelets analysis (frequency, power, and time) or ERP memory-related potentials. Neurofeedback is given by rewarding brain patterns that are associated with accurate and fast memory retrieval.

Existing studies on ERP modulation using NF have revealed insights into designing reward functions. These studies mainly investigate the decoding method of single-trial responses (Brandmeyer et al., 2013) as well as the neural mechanism underling reward stimuli (Moser et al., 2014; Zioga et al., 2019; Wang et al., 2021). Research in the literature demonstrates broad applications of ERP-based NF training in cognitive deficits, including Attention-deficit/hyperactivity disorder (ADHD), Post-traumatic stress disorder (PTSD), and Subjective cognitive decline (SCD) (Askovic et al., 2020; Pei et al., 2020; Deiber et al., 2021). The main challenge of developing NF training using ERP is designing reward functions associated with components of ERP as the adjustment target in single-trial responses. In a recent study, Wang et al. (2021) compared the change of event-related desynchronization (ERD) power attenuation using ERP-based NF vs. motor imagery (MI)-based NF and showed that only ERP based study presents significant differences in the frequency bands of EEG signals between the neurofeedback and non-feedback groups.

## The Summary of Neurofeedback Clinical Trials to Improve Mild Cognitive Impairment

Important for clinical applications of neurofeedback, we reviewed clinical trials related to mild cognitive impairment and dementia using NF training around the world. Table 5 includes studies using only neurofeedback to improve cognitive ability in older adults, in Alzheimer’s disease, and related dementia (AD/ADRD). Importantly, there are different EEG and ERP variation in MCI induced by subtypes of ADRD (Güntekin et al., 2021). Note three times more neurofeedback trials are being conducted to improve mood, depression, and attention at the clinicaltrials.gov site that are not included in the current review.

**TABLE 5:** Summary of clinical trials at clinicaltrials.gov of applying NF training for older adults with MCI.

Name of study	Sex	Age	Recruitment status	Session	Control/sham	Reward protocol	Outcome measures	Key findings
ClinicalTrials.gov, 2019a	All	65–90	Completed	20–70-min over 11 weeks; 2 per week	N/A	12 SMR related video games in the EEG Digitrack system (Marlats et al., 2020)	(1) Rey Auditory Verbal Learning Test (2) SMR related frequency bands of the EEG signal	Not yet reported findings
ClinicalTrials.gov, 2018a	All	> 60	Completed	20–70-min over 11 weeks; 2 per week	N/A	(1) Window size of image (2) Clarity of images and sounds (3) Number of simulated audience member	(1) NF Technology Acceptation questionnaire	Not yet reported findings
ClinicalTrials.gov, 2019b	All	65–90	Unknown	20–40-min over 7 weeks; 2–3 per week	No feedback	(1) Window size of image (2) Clarity of images and sounds (3) Number of simulated audience member	(1) Rey auditory verbal learning test (2) SMR related frequency bands of the EEG signal	Not yet reported findings
ClinicalTrials.gov, 2018b	All	65–90	Unknown	30–30-min over 12 weeks; 2–3 per week	No feedback	(1) Window size of image (2) Clarity of images and sounds (3) Number of simulated audience member	(1) Attention tests, TMTB-TMA (2) Rey Auditory Verbal Learning Test	The NF training protocol could be effective to reduce cognitive deficits in elderly patients with MCI and improve their EEG activity (Marlats et al., 2019, 2020)
ClinicalTrials.gov, 2020c	All	60–80	Recruiting	7 40–80 min over 7 weeks; 1 per week	(1) Healthy elderly participants receiving feedback from the hippocampus (2) Healthy elderly participants receiving feedback from another area (3) Patients with MCI receiving feedback from another brain area	Modify a simulated thermometer with no suggest explicit strategies	(1) Brain activation map with fMRI (2) Behavioral performance in the proposed training	Not yet reported findings
ClinicalTrials.gov, 2016	All	> 50	Unknown	10–60-min over 5 weeks; 2–3 per week	With electrical static activity of a disconnected electrode	Move a ball to the middle of a 3D simulated environment with beeping sound	(1) Upper alpha to lower alpha power ratio and of peak alpha frequency of the EEG signal (2) Cognitive assessments of measuring memory performance and other cognitive domains	The increase of different cognitive domains as well as EEG activity was not preserved at 30 days after training, the improvement in memory was still present. (Lavy et al., 2019, 2021)
ClinicalTrials.gov, 2021b	All	50–85	Recruiting	24–30–45 min over 12 weeks; 2 per week	Random video and music progression which is not depended on brain activities	(1) The video progresses (2) The music continues to play	(1) Gamma frequency band of the EEG signal (2) N-back test	Not yet reported findings
ClinicalTrials.gov, 2022	All	> 60	Not yet recruiting	N/A	Normal healthy Veterans	N/A	(1) Theta frequency band of the EEG signal (2) California Verbal Learning Test	Not yet reported findings
ClinicalTrials.gov, 2021a	All	20–90	Terminated	Daily 5–15 min over 6 weeks	No feedback	The change of weather sound	(1) The average of EEG power spectrum (2) Cognitive assessments for older adults with MCI and caregivers	Not yet reported findings
ClinicalTrials.gov, 2017	All	50–80	Completed	3 60-min over 5 weeks; 1 per week	(1) Healthy older adults (2) Healthy older adults in sham feedback condition	Modify a simulated thermometer with no suggest explicit strategies	(1) Parahippocampal activation as measured with fMRI (2) Cognitive assessments	rtfMRI NF training can improve cognitive abilities in healthy elderly and patients of AD, but these effects may not transfer broadly (Hohenfeld et al., 2017, 2020)
ClinicalTrials.gov, 2020a	All	50–80	Active, not recruiting	N/A	Random subject under sham treatment	Iremember program	Memory, executive function and every fay functionality evaluation	Not yet reported findings
ClinicalTrials.gov, 2020b	All	55–85	Completed	12 weeks	N/A	Memory Boot Camp program	(1) MoCA test	Not yet reported findings

*SMR, sensorimotor rhythms; TMTB-TMTA, trails A and B of the trail making test.*

## The Take-Home Message of Electroencephalography Neurofeedback Studies and Future Directions

Most current Neurofeedback training improves memory and cognition to a certain extent. Large scale and optimal clinical applications of neurofeedback are limited by several challenges. Debate remains about the frequency and length of optimal NF effects, outcome measures, and long-term effects.

A well-known issue in such training is that some people simply do not respond to neurofeedback. Thus, we also reviewed the literature of individual differences in placebo effects and non-responses. Future work needs to focus on an individual based approach.

Sleep EEG has also emerged as a very important biomarker (Whitehurst et al., 2020), especially for outcome measures in neurofeedback studies in the future (Alfini et al., 2020). D’Atri et al. (2021) reported the importance of time of day showing differences in Delta and alpha signals during PM and AM wake studies (lack of difference in patients with cognitive impairment and AD). Rapid Eye Movement (REM) EEG slowing during sleep showed the strongest correlation with cognitive decline than other wake EEG in their study. Sleep EEG also has stable signals. If daytime nap EEG can be managed (Alger et al., 2012), it should be an ideal outcome measure for effectiveness in NF training.

The range of EEG features for NF training are as follows:

– EEG (Various Alpha, frontal theta, sensory-motor rhythm, connectivity during resting-state)
– ERP (Memory-related potentials; event-related connectivity)
– Cognitive impairment due to different pathologies warrants different neurofeedback, e.g., AD vs. Non-AD (LB dementia)
– Higher EEG rhythm such as gamma frequency is gaining attention.
– EEG features during sleep for memory enhancement during sleep and as outcome measures.

## Conclusion

Our review brings overall good news of the potential effectiveness of neurofeedback brain training. Most training studies typically take multiple weeks (e.g., 5–20 weeks) with 2–3 sessions per week. We review various neurofeedback reward strategies (visual and auditory methods) and outcome measures. Our review recognizes that individuals’ neural responses are on a continuous spectrum. We recommend that “neural modulation sensitivity” instead of “BCI illiteracy” is to be considered as the preferred terminology in brain training. Future directions include much needed research in mild cognitive impairment, in non-Alzheimer’s dementia populations, and neurofeedback using EEG features during resting and sleep for memory enhancement and as sensitive outcome measures.

## Author Contributions

YJ wrote the first draft and oversaw all aspects of the revisions. WJ wrote the reviews summarized in Tables 1, 2. SH wrote the reviews summarized in Tables 3, 4. SB wrote the closed-loop real-time NF section. ZL wrote the related review summarized in Table 5. XZ conceptualized and wrote the BCI portion. LP and WH contributed to conceptualization and interpretation of EEG-based NF training. JS and SC-S contributed to conceptualization of clinical implications, critical revision, overall edits, and to Figure 1.

## Conflict of Interest

The authors declare that the research was conducted in the absence of any commercial or financial relationships that could be construed as a potential conflict of interest.

## Publisher’s Note

All claims expressed in this article are solely those of the authors and do not necessarily represent those of their affiliated organizations, or those of the publisher, the editors and the reviewers. Any product that may be evaluated in this article, or claim that may be made by its manufacturer, is not guaranteed or endorsed by the publisher.
